# Predicting Adverse Pathologic Features and Clinical Outcomes of Resectable Pancreas Cancer With Preoperative CA 19-9

**DOI:** 10.3389/fonc.2021.651119

**Published:** 2021-05-11

**Authors:** Roman O. Kowalchuk, Scott C. Lester, Rondell P. Graham, William S. Harmsen, Lizhi Zhang, Thorvardur R. Halfdanarson, Rory L. Smoot, Hunter C. Gits, Wen Wee Ma, Dawn Owen, Amit Mahipal, Robert C. Miller, Michelle A. Neben Wittich, Sean P. Cleary, Robert R. McWilliams, Michael G. Haddock, Christopher L. Hallemeier, Mark J. Truty, Kenneth W. Merrell

**Affiliations:** ^1^ Department of Radiation Oncology, Mayo Clinic, Rochester, MN, United States; ^2^ Department of Pathology, Mayo Clinic, Rochester, MN, United States; ^3^ Department of Statistics, Mayo Clinic, Rochester, MN, United States; ^4^ Department of Medical Oncology, Mayo Clinic, Rochester, MN, United States; ^5^ Department of Pancreas Surgery, Mayo Clinic, Rochester, MN, United States

**Keywords:** pancreatic cancer, CA19-9, prognostic factors, neoadjuvant, resection

## Abstract

**Background:**

We evaluated preoperative CA 19-9 levels in patients with resected pancreatic cancer to analyze whether they were predictive of clinical outcomes and could help select patients for additional therapy. We hypothesized that elevated CA 19-9 would be associated with worse pathologic findings and oncologic outcomes.

**Methods:**

This study assessed 509 patients with non-metastatic pancreatic adenocarcinoma who underwent resection at our institution from 1995-2011 and had preoperative CA 19-9 recorded. No patients received neoadjuvant therapy. CA 19-9 level was analyzed as a continuous and a dichotomized (> *vs*. ≤ 55 U/mL) variable using logistic and Cox models.

**Results:**

Median follow-up was 7.8 years, and the median age was 66 years (33-90). 64% of patients had elevated preoperative CA 19-9 (median: 141 U/mL), that did not correlate with bilirubin level or tumor size. Most patients had ≥ T3 tumors (72%) and positive lymph nodes (62%). The rate of incomplete (R1 or R2) resection was 19%. Increasing preoperative CA 19-9 was associated with extra-pancreatic extension (p=0.0005), lymphovascular space invasion (p=0.0072), incomplete resection [HR (95% CI) 2.0 (1.2-3.5)], and lower OS [HR = 1.6 (1.3-2.0)]. Each doubling in preoperative CA 19-9 value was associated with an 8.3% increased risk of death [HR = 1.08 (1.02-1.15)] and a 10.0% increased risk of distant recurrence [HR = 1.10 (1.02-1.19)]. Patients classified as non-secretors had comparable outcomes to patients with normal CA 19-9.

**Conclusions:**

Elevated preoperative CA 19-9 level was associated with adverse pathologic features, incomplete resection, and inferior clinical outcomes. Neither tumor size nor bilirubin confound an elevated CA 19-9 level. Preoperative CA 19-9 level may help select patients for additional therapy.

## Introduction

Pancreatic ductal adenocarcinoma (PDAC) represents the fourth leading cause of cancer death in the United States (1). The only treatment associated with potential cure is complete resection; however, only 20% of the patients have anatomically resectable disease according to standard criteria at the time of diagnosis (2). Based on adjuvant therapy trials, up to 60% of these patients will have an incomplete resection with positive microscopic (R1) or gross (R2) residual disease, negating any oncologic benefit from resection (3–5). Even for patients with complete resection, the rates of locoregional (LR) and distant relapse (DR) remain high (6). Clinical trials support the standard role of adjuvant chemotherapy though routine use of adjuvant radiation therapy remains controversial (3–5, 7–13). Several recent clinical trials show that neoadjuvant chemotherapy followed by chemoradiation may improve the rates of R0 resection, lymph node sterilization, local control, and possibly overall survival (OS) compared to upfront resection, with benefits likely being greatest for patients with borderline resectable pancreas cancer (6, 14–16). The phase III randomized PREOPANC study suggested that preoperative chemoradiotherapy may lead to tumor downstaging and a reduction in the rates of adverse pathologic features (16). The ideal selection process and factors to decide between upfront surgical versus a neoadjuvant approach are not yet optimized in patients with otherwise anatomically resectable tumors.

CA 19-9 is a Lewis blood group antigen that is measurable in up to 90% of patients, with approximately 10% of patients lacking the fucosyl-transferase necessary for expression (i.e. non-secretors). Up to two-thirds of patients have elevated CA 19-9 levels at presentation; however, these levels may also be falsely elevated due to concomitant biliary obstruction at diagnosis (17, 18). Previous studies have assessed preoperative CA 19-9 levels and suggest elevated CA 19-9 levels predict for higher tumor stage, adverse pathologic features, and tumor resectability in upfront resectable pancreas cancer patients (19–22). In addition, elevated CA 19-9 can predict worse survival outcomes (19, 23–28). An NCDB analysis of over 100,000 pancreatic cancer patients concluded that any elevation of CA 19-9 above normal was associated with a significant survival detriment, greatest in early stage resectable disease. Neoadjuvant chemotherapy was the only treatment strategy able to eliminate this survival detriment in anatomically resectable pancreatic cancer, more so even than adjuvant chemotherapy (29). Serial measures of CA 19-9 may predict tumor biology and response to therapy in locally advanced disease, and an early decrease in CA 19-9 following administration of gemcitabine is associated with improved overall survival (30). Multidisciplinary care is critical to optimizing treatment approaches for patients with pancreatic cancer, and further clarification of the predictive and prognostic value of CA 19-9 will assist healthcare teams with optimizing therapy for resectable pancreatic cancer (31, 32).

In the present study, we evaluated a large cohort of patients to assess the association of preoperative elevated CA 19-9 level with adverse pathologic features and clinical endpoints. In the era of neoadjuvant therapy for pancreas cancer, we aim to evaluate the potential of preoperative CA 19-9 level as a key tool to optimize patient selection for neoadjuvant therapy.

## Methods

### Patients

This retrospective study was approved by our institutional review board (IRB: 17-003122). As a minimal risk IRB, a HIPPA waiver was used, and informed consent was not required. The institutional cancer registry was queried for all patients with non-metastatic pancreatic adenocarcinoma from March 1995 to January 2011. Treatments over this timeframe allowed for a relatively homogeneous assessment in the pre-neoadjuvant era. Additionally, prolonged follow-up was available for this set of patients. 1140 patients were reviewed in total, with 631 patients excluded. Of these, 451 had no CA 19-9 available, 104 had inadequate follow-up, 50 had surgical resection at an outside institution, and 26 had neoadjuvant treatment. This resulted in the final study cohort of 509 patients available for full evaluation.

### CA 19-9 Level Measurement

Serum CA 19-9 level was measured using an immunoenzymatic sandwich assay. A Bayer/Siemens ACS 180 chemistry analyzer (Siemens Healthcare Diagnostics Inc, Deerfield, IL) was utilized from 1995 to July of 2001. From July 2001 to 2005, a Bayer/Siemens Centaur (Siemens Health Siemens Healthcare Diagnostics Inc, Deerfield, IL) instrument was used. After 2005, the DXI 800 (Beckman Coulter Inc, Chaska, MN) was utilized for CA 19-9 level measurement. A threshold of 55 U/mL was used as the institutional reference for a normal CA 19-9 level during the period of patient inclusion, and elevated CA 19-9 level was defined as above this institutional reference. Patients without measurable CA 19-9 levels were considered non-secretors.

### Patient Treatment

All patients underwent curative intent resection in an upfront surgical approach, without any preoperative therapy. Resection types included pancreaticoduodenectomy, distal pancreatectomy, and total pancreatectomy. Pathologic features evaluated include TNM stage, the ratio of positive lymph nodes to total dissected (LNR), resection margin status, perineural and lymphovascular space invasion (LVSI), tumor grade, and tumor size. Staging was assessed using the AJCC 7^th^ edition. For this reason, comparisons were made between T1 or T2 tumors, compared with T3 or T4 tumors (those demonstrating extra-pancreatic extension). In this study, an R0 resection was defined as a resection achieving negative gross and microscopic margins within 1 mm of the inked margin. After surgery, patients received adjuvant therapy per the discretion of the multidisciplinary treatment team. Adjuvant therapy consisted of chemotherapy alone, chemoradiation or combination chemotherapy and chemoradiation. Radiation was delivered with concurrent chemotherapy, most frequently 5-flourouracil or capecitabine.

### Assessment of Relapse

LR was defined as local (tumor bed or remnant pancreas) or regional (regional lymph node) disease progression based on imaging. DR was defined as distant organ or distant lymph node spread consistent with metastatic disease. When available, CA 19-9 level, biopsy, and surgical results were used to corroborate radiographic findings of relapse. The presence of LR or DR was assessed until the time of death. OS was defined from the surgical date until patient death or last follow up. To minimize selection bias, patients with less than 90 days of survival after surgery, those with R2 resection, or patients who did not receive adjuvant therapy were excluded from the survival and oncology outcome analysis.

### Statistics

Logistic regression and Spearman’s rank correlation were used to assess the relationship between CA 19-9 levels and pathologic outcomes. The Spearman rank test was used rather than the Pearson test due to the wide range and right-skew of the data. The Kaplan Meier method was used to estimate survival free of DR and OS from the time of surgery to recurrence or death. For LR, the cumulative incidence was estimated considering death as a competing risk. Univariate Cox proportional hazard models were used to evaluate associations between CA 19-9 level and adverse pathology features with OS and DR. For LR, the Fine and Gray extension of the Cox model was used. The backward selection process was used to identify variables for inclusion in the multivariable model. Patients classified as non-secretors were only included in survival analysis. A p-value < 0.05 was considered statistically significant.

## Results

### Patient Characteristics

**Figure 1:**
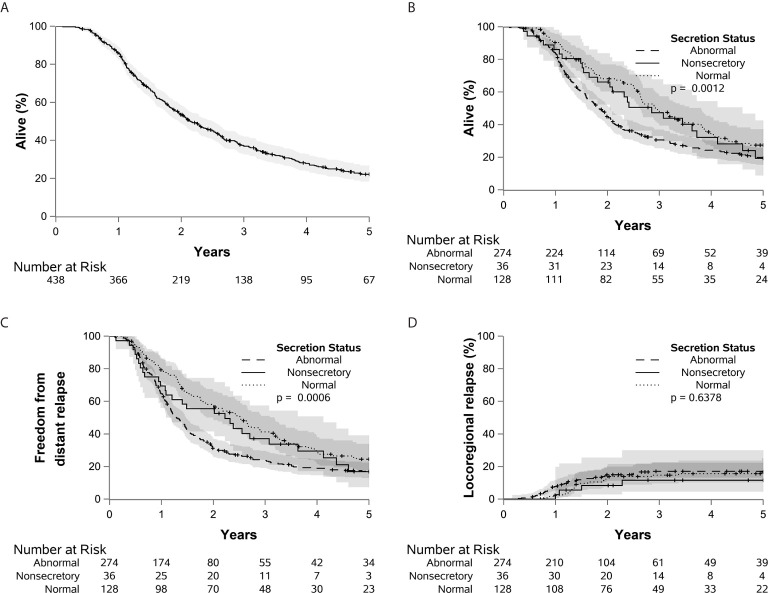
Kaplan-Meier curves are shown demonstrating the overall survival of the entire cohort **(A)**, as well as overall survival **(B)**, distant relapse **(C)**, and locoregional relapse **(D)** stratified by CA 19-9 status.

A total of 509 patients were evaluated and included in the study. With a median follow up of 7.8 years (range, 0.1-16 years), 21% of patients remained alive at the time of last follow-up. Patient and tumor characteristics are reported in **Table 1**. A total of 324 patients (64%) had elevated preoperative CA 19-9; 138 patients (27%) had normal CA 19-9; and 47 (9%) were found to be non-secretors. The median preoperative CA 19-9 value was 140.5 U/mL (range, 4 to 335,300 U/mL). For all patients, the median OS was 2.0 years and the 5-year OS was 21.6% (95% CI 18%-26%) (**Figure 1A**). Gemcitabine was the most frequently administered adjuvant chemotherapy, with a median 6 of cycles given. The median radiation dose was 50.4 Gy in 28 fractions (range 45-55 Gy).

**Table 1:** Patient characteristics are tabulated.

	All Patients
Age, mean (SD)	65 (10.9)
Gender, n (%)	
Male	276 (54.2)
Female	233 (45.9)
Performance Status, mean (SD)	0.2(0.4)
Charlson Index, median (range)	4 (0-11)
Histology, n (%)	
Adenocarcinoma/Infiltrating Duct	496 (96.1)
Mucinous carcinoma	11 (2.1)
Other	9 (1.8)
Tumor Site, n (%)	
Head	405 (79.6)
Body	32 (6.3)
Tail	32 (6.3)
Duct	4 (0.8)
Other	6 (1.2)
Overlap	10 (2.0)
NOS	20 (3.9)
Tumor Size, mean (SD)	38.4 (61.6)
Pathologic T stage, n (%)	
T1	32 (6.3)
T2	114 (22.0)
T3	342 (67.2)
T4	23 (4.5)
Pathologic N stage, n (%)	
N0	192 (37.7)
N1	317 (62.3)
Resection Margin, n (%)	
R0	411 (80.7)
R1	82 (16.1)
R2	16 (3.1)
Tumor Grade, n (%)	
1	3 (0.6)
2	69 (13.6)
3	374 (73.5)
4	63 (12.4)
Adjuvant Treatment	
None	55 (10.8)
Chemoradiation	171 (33.6)
Chemotherapy then chemoradiation	206 (40.5)
Chemotherapy	74 (14.5)
Unknown	1 (0.2)
Immunotherapy then chemoradiation then chemotherapy	2 (0.4)

### Preoperative CA 19-9 and Adverse Pathologic Features

**Figure 2:**
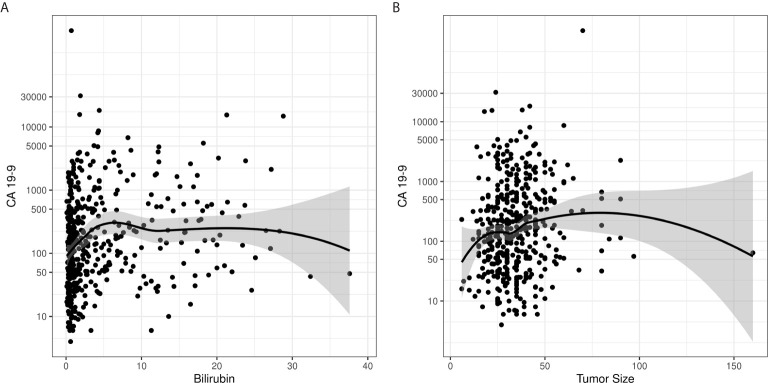
Loess curves demonstrate a lack of correlation between CA 19-9 levels and **(A)** bilirubin and **(B)** tumor size.

Preoperative CA 19-9 level was analyzed as a continuous and a dichotomized value (elevated *vs*. normal). For patients with multiple recorded preoperative CA 19-9 levels, the last value drawn before surgery was used. For these analyses, CA 19-9 non-secretors were excluded to prevent skewing of the resulting analysis. We found that serum CA 19-9 levels did not correlate with either serum bilirubin or tumor size (**Figure 2**). Using a rank sum test of continuous CA 19-9 value, higher CA 19-9 levels were associated with T stage ≥ 3 *vs*. < 3 (p=0.0005, median 172 *vs*. 94), LVSI present *vs*. absent (p=0.0072, median 224 vs. 130), and LNR > 7% vs. ≤ 7% (p=0.048, median 167 *vs*. 115) (**Table 2**). Increasing levels of CA 19-9 were correlated with increases in the number of positive nodes (p=0.004, correlation coefficient 0.14), LNR (p=0.0005, correlation coefficient 0.16), and tumor size (p=0.002, correlation coefficient 0.15) when considered as a continuous variable.

**Table 2:** The association of CA 19-9 and adverse pathologic features among patients with normal and elevated CA 19-9 is demonstrated.

	Median Two-Sample Test		
	N	Median CA 19-9 (IQR)	P-value^†^
T stage			
3 or 4	333	172 (52-593)	**0.0005**
1 or 2	129	94 (35-230)	
N stage			
N1	289	162 (53-522)	0.070
N0	173	103 (43-438)	
LVSI			
Yes	59	224 (59-1019)	**0.0072**
No	409	131 (46-467)	
Perineural Invasion			
Yes	137	163 (52-509)	0.71
No	324	130 (46-496)	
Missing	1	895 (895-895)	
Grade			
3 or 4	400	149 (51-496)	0.46
1 or 2	62	113 (43-542)	
Resection Margin			
R1/R2	93	213 (67-603)	**0.011**
R0	369	131 (45-438)	

N, number; IQR, interquartile range; OR, odds ratio; LVSI, lymphovascular space invasion; LNR, lymph node ratio.

^†^Significance test using Wilcoxon rank sum test. Bold values indicate those that reached statistical significance.

CA 19-9 was analyzed as a dichotomous variable in two separate analyses, one in which the non-secretor groups was combined with the patients with normal CA 19-9 levels, and one in which the non-secretors, normal CA 19-9 level patients, and elevated CA 19-9 level patients were separated into 3 distinct groups. Elevated CA 19-9 (> 55 U/mL) was then analyzed as a predictor of T stage 3 or 4, node positivity, LVSI, perineural invasion, grade 3 or 4, tumor size ≥ 3.5 cm, and incomplete resection. Elevated CA 19-9 as a dichotomized variable only reached statistical significance for incomplete resection in the two and three group settings (p=0.007 and p=0.0047, respectively).

### Preoperative CA 19-9 and Resection Margins

The overall rate of R1/R2 resection was 19%. The median CA 19-9 values for patients with R0, R1 and R2 resections were 108 U/mL (interquartile range (IQR), 28-361), 154 U/mL (IQR, 53-543), and 642 U/mL (IQR, 185-2135). Using a Kruskal Wallis test, CA 19-9 level was significantly different for patients with R0, R1, and R2 resections (p=0.003). The Wilcoxon rank sum test showed statistically higher levels of CA 19-9 was associated with R1 resections, compared to R0 (p=0.0009). Patients with a normal CA 19-9 level had a rate of incomplete resection (R1 or R2) of 12.4% *vs*. 22% for patients with elevated CA 19-9. Similarly, logistic regression analysis showed an association between abnormal CA 19-9 level and incomplete resection [p=0.01, HR=2.0 (1.2-3.5)].

### Preoperative CA 19-9 and Relapse and Survival

At the time of evaluation, there were a total of 404 patient deaths. This included 33 patients (70%) in the non-secretor group, 101 patients (73%) in the normal CA 19-9 group, and 270 patients (83%) in the elevated CA 19-9 group. Prior to further analysis of the outcomes, 55 patients without adjuvant therapy, 16 with R2 resection, and one with death within 90 days of surgery were excluded. The 5-year OS was 19% (95% CI, 9%-43%), 27% (95% CI, 20%-37%), and 20% (95% CI, 15%-26%) for the non-secretors, normal and elevated CA 19-9 groups respectively (p=.0012) (**Figures 1B–D**). Relative to patients with normal CA 19-9, elevated CA19-9 was associated with a statistically significant decrease in OS [p=0.0015, HR=1.49 (1.17-1.90)] and an increased risk of DR [p=0.0121, HR=1.45 (1.09-1.93)]. Elevated CA19-9 was also associated with decreased freedom from death or DR [p<0.001, HR=1.57 (1.24-1.99)] (**Supplementary Figure 1**). These values are reported using the parsimonious model, retaining only variables where p < 0.05 using backward selection. Elevated CA 19-9 was not associated with risk of LR [p=0.69, HR=1.11 (0.66-1.87)]. There was no significant difference between the normal CA 19-9 group and the non-secretor group in regards to OS, DR, or LR.

**Figure 3:**
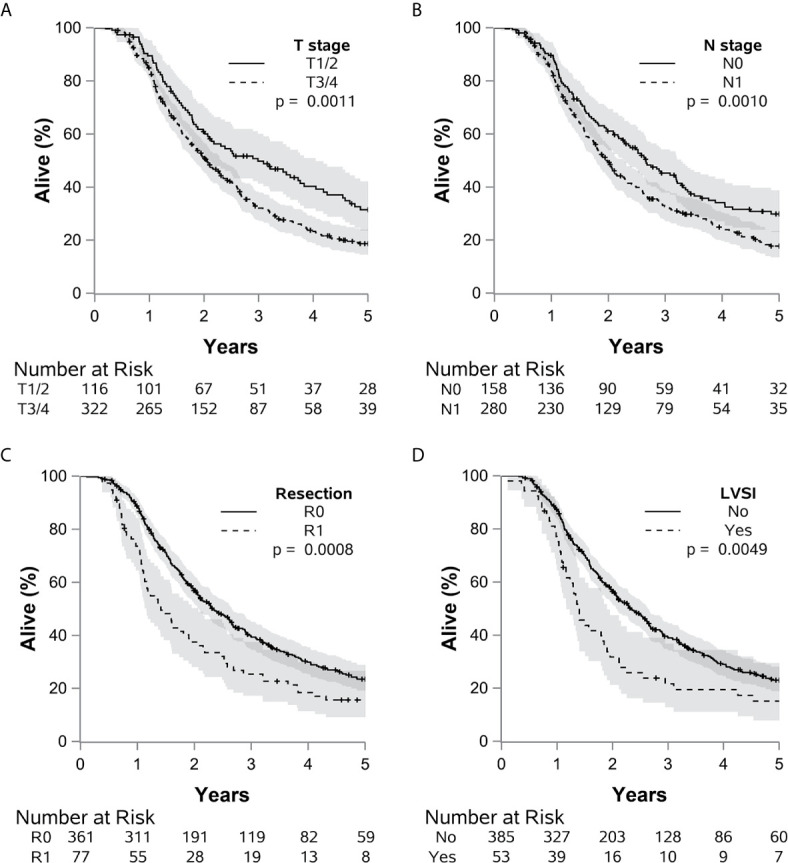
The impact of many adverse pathologic factors on survival is portrayed, with **(A)** T 3 or 4 vs. 1 or 2 (p=0.0011), **(B)** N1 vs. N0 (p=0.0010), **(C)** R1 vs. R0 (p=0.0009), and **(D)** LVSI present vs. absent (p=0.0049) all reaching statistical significance. Grade 4 or 3 vs. 1 or 2 (p=0.061) demonstrated a potential statistical trend.

When analyzed as a continuous variable using linear association with log base 2 of the preoperative CA 19-9 value, each doubling in CA 19-9 value was associated with an 8.3% increased risk of death [p<0.0001, HR=1.08 (1.02-1.15)], a 10.0% increased risk of DR [p=0.006, HR=1.10 (1.02-1.19)]. For this analysis, patients classified as non-secretors were excluded. Adverse pathologic features that were associated with elevated CA 19-9 were also found to have a negative impact on patient survival (**Figure 3**). This analysis was conducted with univariate and multivariate Cox analyses (**Table 3**). Adjuvant chemotherapy along (without radiotherapy) was also associated with decreased overall survival [p=0.0019, HR=1.60 (1.19-2.16)] and LR [p<0.0001, HR=3.07 (1.84-5.12)] in the overall cohort.

**Table 3:** Multiple variable (MVA) and univariate Cox analyses for overall survival (OS), freedom from distant recurrence (DR), and loco-regional disease progression (LR) are shown.

Variable	OS: univariate	OS: MVA	DR: univariate	DR: MVA	LR: univariate	LR: MVA
Pre-op CA 19-9						
Abnormal	**1.53 (1.20-2.00)**	**1.45 (1.13-1.85)**	**1.35 (1.01-1.79)**	**1.43 (1.08-1.89)**	1.11 (0.66-1.87)	1.43 (0.86-2.37)
Nonsecretory	1.05 (0.68-1.62)	1.07 (0.69-1.66)	1.17 (0.70-1.97)	1.17 (0.66-2.08)	0.70 (0.24-2.01)	0.79 (0.26-2.39)
Normal	Reference	Reference	Reference	Reference	Reference	Reference
T stage						
3/4	**1.51 (1.18-1.94)**	**1.30 (1.00-1.68)**	1.23 (0.92-1.65)	1.13 (0.82-1.54)	0.99 (0.59-1.67)	1.10 (0.64-1.88)
1/2	Reference	Reference	Reference	Reference	Reference	Reference
N stage						
N1	**1.47 (1.17-1.84)**	**1.31 (1.03-1.66)**	**1.42 (1.08-1.88)**	1.33 (0.99-1.79)	1.22 (0.74-2.03)	1.52 (0.87-2.65)
N0	Reference	Reference	Reference	Reference	Reference	Reference
Margin status						
R1	**1.57 (1.21-2.05)**	**1.49 (1.10-1.91)**	1.31 (0.95-1.81)	**1.39 (1.01-1.91)**	0.68 (0.33-1.39)	0.87 (0.43-1.78)
R0	Reference	Reference	Reference	Reference	Reference	Reference
LVSI						
Present	**1.56 (1.14-2.12)**	**1.38 (1.01-1.88)**	**1.68 (1.16-2.42)**	**1.67 (1.17-2.40)**	1.10 (0.54-2.23)	1.36 (0.68-2.71)
None	Reference	Reference	Reference	Reference	Reference	Reference
Grade						
3/4	1.38 (0.99-1.94)	1.27 (0.90-1.80)	**1.85 (1.21-2.82)**	**1.88 (1.21-2.91)**	1.23 (0.57-2.64)	1.28 (0.57-2.88)
1/2	Reference	Reference	Reference	Reference	Reference	Reference
Tumor size, per 10 mm	**1.06 (1.01-1.11)**	1.05 (1.00-1.11)	1.05 (0.99-1.11)	1.06 (1.00-1.13)	0.96 (0.85-1.07)	0.98 (0.89-1.08)
Adjuvant						
Chemo alone	**1.44 (1.07-1.93)**	**1.60 (1.19-2.16)**	1.19 (0.85-1.68)	1.41 (1.00-1.99)	**2.73 (1.65-4.53)**	**3.23 (1.93-5.40)**
Chemoradiation	Reference	Reference	Reference	Reference	Reference	Reference

MVA, multiple variable analysis; OS, overall survival; DR, freedom from distant recurrence; LR, loco-regional disease progression; LVSI, lymphovascular space invasion. Hazard ratios from the initial multiple variable model are reported (prior to backward selection). Bold values indicate those that reached statistical significance.

## Discussion

This large, single institutional study with long-term follow-up of patients with localized pancreas cancer treated with an upfront surgical approach demonstrates the utility of pre-operative CA 19-9 level in predicting adverse pathologic features, resection margin outcomes. In agreement with previously reported series, elevated pre-operative CA 19-9 level was associated with higher risk pathologic features, including T stage, multiple lymph node involvement, and LVSI (19, 21, 33, 34). We show these adverse pathologic features associated with CA 19-9 elevation are also predictive of patient survival, further reinforcing the link between elevated CA 19-9, advanced cancer stage, and subsequently worse patient outcomes (21, 28, 35–40). Additionally, there was no association between CA 19-9 levels and serum bilirubin levels or tumor size. This analysis constitutes the first robust demonstration that these potential confounders do not significantly influence CA 19-9 level; therefore, any CA 19-9 elevation may be biologically relevant, despite the presence of potential confounders.

The association between elevated CA 19-9 level and increased rates of R1 and R2 resections is a critical finding because achieving R0 resection is required to potentially cure pancreas cancer (41, 42). In the present study, patients with elevated CA 19-9 levels were twice as likely to have positive margins as patients with normal CA 19-9 levels. CA 19-9 level was predictive of increased rates of incomplete resection both as a continuous variable and as a dichotomized value with a threshold of 55 U/mL, the reference value for normal during the period of this study. Other studies have posited that higher CA 19-9 values might predict for unresectable disease, but they have generally been smaller analyses (20). Hartwig et al. reported findings from their analysis of 1,626 consecutive patients who underwent upfront surgical resection for primary pancreatic adenocarcinoma, and they noted R0 rates as low as 15% in patients with CA 19-9 levels ≥ 1000 U/mL (43). This study importantly also found a continuous decrease in the R0 resection rate and subsequent survival with increased values of CA 19-9, and multivariate analysis found the CA 19-9 level to be the most valuable independent predictor for resectability.

The standard reference of “normal CA 19-9” has changed over the years dependent on specific institutional assays, making direct comparisons between specific upper limits of CA 19-9 difficult. Although multiple centers have published different CA 19-9 cutoffs, none of these thresholds are clinically utilized to decide between upfront surgery or neoadjuvant therapy (22, 35, 40). Coupled with the loss of significance of CA 19-9’s predictive power for the many pathologic factors when analyzed as a dichotomized variable, as opposed to as a continuous variable, these findings suggest that a simple threshold is an insufficient way to assess CA 19-9 level.

In the present study, we show an 8.3% increased risk of death and a 10.0% increased risk of DR with each doubling of CA 19-9 level. This association between elevated preoperative CA 19-9 level and decreased OS is consistent with the literature, and our result reinforces the prognostic value of this test (43, 44). These results correlate with the findings of Mattiuci et al., who reported a graded decrease in 5-year OS using 4 separate CA 19-9 cutoffs (45).

Coupled with our findings in this study, these results suggest that any elevation of CA 19-9 above reference level should be considered an adverse risk feature, with higher elevations portending decreased rates of resectability and subsequent survival with an upfront, surgery-first approach. Thus, patients presenting with any CA 19-9 level elevation should be strongly considered for neoadjuvant therapy, regardless of anatomic resectability. The association of CA 19-9 level with numerous adverse pathologic features and decreased survival supports the idea that these patients may benefit from neoadjuvant therapy. Additional justification includes the fact that patients had decreased OS (HR=1.60 [1.12-2.16]) with adjuvant chemotherapy alone, compared to chemoradiation. Administration of neoadjuvant chemotherapy and chemoradiation presents the opportunity to (1) downstage unresectable or borderline resectable patients, (2) detect occult metastatic disease in patients likely to develop regional or distant progression in the immediate post-operative period, and (3) enhance the tolerance of and ability to complete all intended therapies that may otherwise be in jeopardy due to post-operative complications or performance status changes.

There is growing evidence showing the benefit of neoadjuvant therapy prior to resection in the management of pancreas cancer. For example, the R0 resection rates with neoadjuvant chemoradiotherapy were 71% *vs*. 40% for upfront surgery in patients enrolled on the PREOPANC clinical trial, inclusive of both resectable and borderline-resectable pancreas cancer (16). Further, adverse pathologic features such as pathologic lymph nodes and LVSI were less common in patients who received neoadjuvant chemoradiation. In addition to assessing a patient’s response to chemoradiotherapy, the association of preoperative CA 19-9 level with adverse pathologic features and subtotal resection may aid in selecting the optimal timing of resection (23, 46, 47). Future studies are needed to better understand the kinetics of CA 19-9 level changes with neoadjuvant therapy, and additional biomarkers would help risk stratify patient who are CA 19-9 non-secretors (48). Lastly, it should be noted that other critical factors, such as radiographic findings of vascular involvement, assessments of a patient’s performance status, and recommendations from multidisciplinary tumor boards, should also be carefully considered. We suggest that the best practice recommendation is for all patients with elevated CA 19-9 level to be considered for neoadjuvant or other additional therapy.

The chief change over the time period of this study (1995 to 2011) involved the different technology used to detect CA 19-9. The impact of the resulting variance would likely be quite small, especially because CA 19-9 was also analyzed as a continuous variable. There was also evidence of changes in practice patterns, chiefly consisting of a decline of adjuvant chemoradiation in favor of chemotherapy followed by chemoradiation. Patients generally had similar rates of adjuvant treatment, overall. This cohort also excluded patients who were found to be unresectable at the time of surgery because emphasis was placed on assessment of patients who underwent resection. It is likely that these patients had generally higher CA 19-9 levels and, therefore, could be candidates for inclusion in future studies. Finally, the retrospective nature of this analysis carries inherent biases, which somewhat limit the scope of the conclusions.

## Conclusions

Elevated and increasing preoperative CA19-9 levels were associated with adverse pathologic features, incomplete resection, and inferior clinical outcomes and not associated with potential confounders, like tumor size or serum bilirubin. Preoperative CA 19-9 level may aid in patient selection for neoadjuvant therapy, but further investigation and validation is warranted.

## Data Availability Statement

The raw data supporting the conclusions of this article will be made available by the authors, without undue reservation.

## Ethics Statement

The studies involving human participants were reviewed and approved by the Mayo Clinic Institutional Review Board. Written informed consent for participation was not required for this study in accordance with the national legislation and the institutional requirements.

## Author Contributions

RK: writing and methodology. SL: writing. WH: formal analysis and methodology. MT: methodology. KM: conceptualization, data curation, and writing. All authors: review and editing. All authors contributed to the article and approved the submitted version.

## Conflict of Interest

RM is the Editor in Chief of ASTRO Advances in Radiation Oncology.

The remaining authors declare that the research was conducted in the absence of any commercial or financial relationships that could be construed as a potential conflict of interest.

## Supplementary Material

Supplementary Figure 1 The association between death or distant relapse and CA 19-9 is shown. Abnormal CA 19-9 was associated with decreased freedom from death or distant relapse (p<0.001).
