# Supplementary figures and images for: Predicting Adverse Pathologic Features and Clinical Outcomes of Resectable Pancreas Cancer With Preoperative CA 19-9
Source: Front Oncol. 2021 May 11;11:651119. doi: 10.3389/fonc.2021.651119 (PMC8147692; doi:10.3389/fonc.2021.651119)

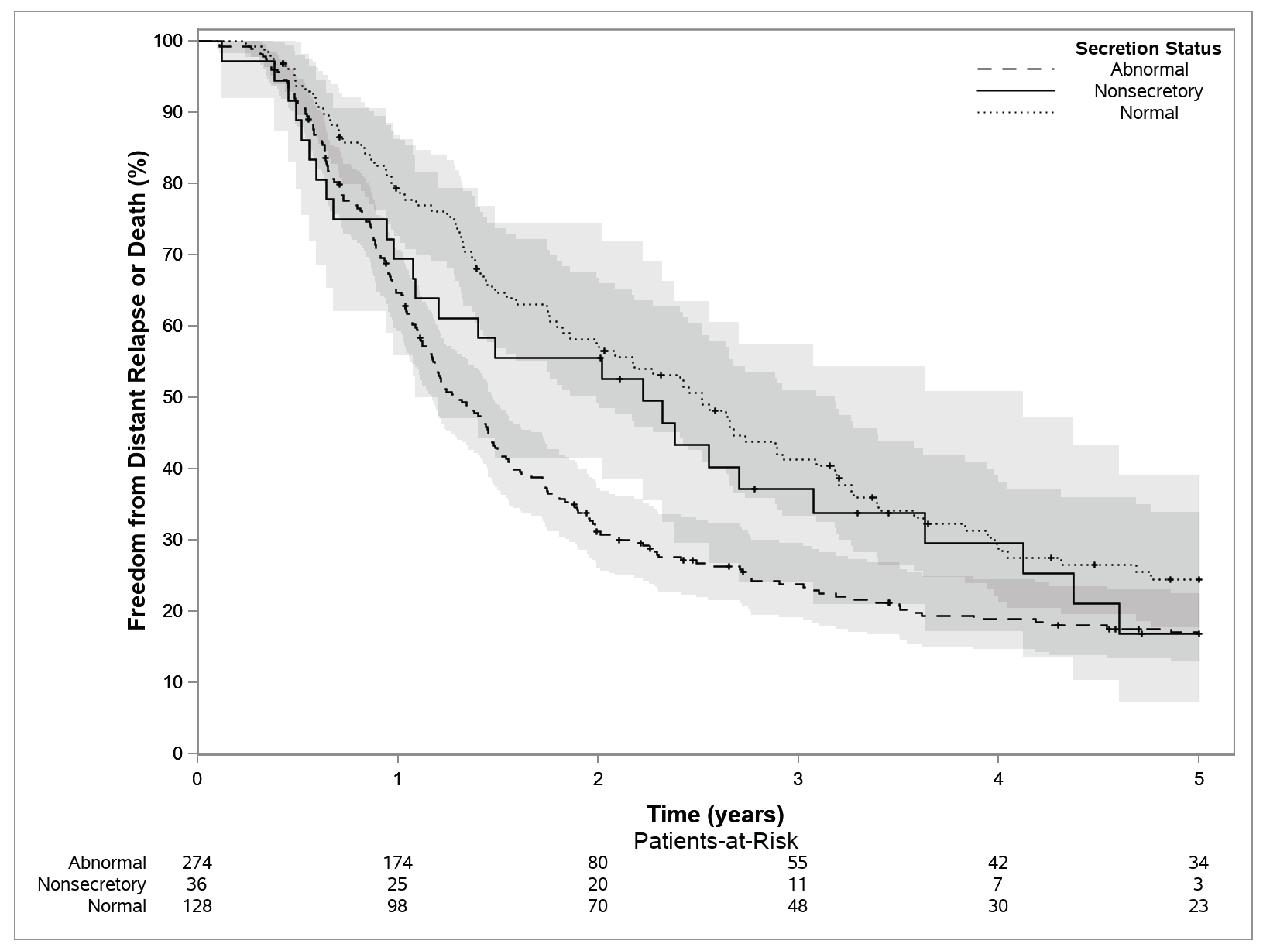

Supplement: Supplementary Figure 1 — The association between death or distant relapse and CA 19-9 is shown. Abnormal CA 19-9 was associated with decreased freedom from death or distant relapse (p<0.001). [file Image_1.tif]
